# In Silico Analysis of Pathogenic *CRB1* Single Nucleotide Variants and Their Amenability to Base Editing as a Potential Lead for Therapeutic Intervention

**DOI:** 10.3390/genes12121908

**Published:** 2021-11-27

**Authors:** Julia-Sophia Bellingrath, Michelle E. McClements, Maria Kaukonen, Manuel Dominik Fischer, Robert E. MacLaren

**Affiliations:** 1 Nuffield Laboratory of Ophthalmology, Nuffield Department of Clinical Neurosciences, University of Oxford, Oxford OX3 9DU, UK; sophia.bellingrath@ndcn.ox.ac.uk (J.-S.B.); michelle.mcclements@eye.ox.ac.uk (M.E.M.); maria.kaukonen@ndcn.ox.ac.uk (M.K.); dominik.fischer@eye.ox.ac.uk (M.D.F.); 2 Oxford Eye Hospital, Oxford University Hospitals NHS Foundation Trust, Headley Way, Oxford OX3 9DU, UK

**Keywords:** *CRB1*, inherited retinal disease, genetics, base editing, CRISPR

## Abstract

Mutations in the *Crumbs homolog 1* (*CRB1*) gene cause both autosomal recessive retinitis pigmentosa (RP) and Leber congenital amaurosis (LCA). Since three separate CRB1 isoforms are expressed at meaningful levels in the human retina, base editing shows promise as a therapeutic approach. This retrospective analysis aims to summarise the reported pathogenic *CRB1* variants and investigate their amenability to treatment with currently available DNA base editors. Pathogenic single nucleotide variants (SNVs) were extracted from the Leiden open-source variation database (LOVD) and ClinVar database and coded by mutational consequence. They were then analyzed for their amenability to currently available DNA base editors and available PAM sites from a selection of different Cas proteins. Of a total of 1115 unique CRB1 variants, 69% were classified as pathogenic SNVs. Of these, 62% were amenable to currently available DNA BEs. Adenine base editors (ABEs) alone have the potential of targeting 34% of pathogenic SNVs; 19% were amenable to a CBE while GBEs could target an additional 9%. Of the pathogenic SNVs targetable with a DNA BE, 87% had a PAM site for a Cas protein. Of the 33 most frequently reported pathogenic SNVs, 70% were targetable with a base editor. The most common pathogenic variant was c.2843G>A, p.Cys948Arg, which is targetable with an ABE. Since 62% of pathogenic *CRB1* SNVs are amenable to correction with a base editor and 87% of these mutations had a suitable PAM site, gene editing represents a promising therapeutic avenue for *CRB1*-associated retinal degenerations.

## 1. Introduction

The field of genome engineering was revolutionised by the harnessing of the bacterial immune system CRISPR/Cas to create programmable DNA double strand breaks in the human genome with an unprecedented ease and adaptability [1]. Unlike traditional CRISPR/Cas systems that induce targeted double stranded DNA breaks (DSB) and require a donor template for mutation correction, base editors harness the DNA binding abilities of the CRISPR/Cas system without the endonuclease activity, thus enabling direct, irreversible chemical modification of single target nucleobases in the genome or transcriptome without creating DSB [2]. Base editors harness naturally occurring deaminases, such as the cytidine deaminase APOBEC1 or *Escherichia coli’s* adenine deaminase TadA (ecTadA) and link them to partially or fully deactivated CRIPSR associated (Cas) proteins such as *Streptococcus pyogenes’* Cas9 (SpCas9). Each base editor requires a 20 nucleotide (nt) single guide RNA (gRNA) which binds to the DNA strand complementary to the target nucleobase and allows for RNA-guided, programmable base editing. In 2016, Komor et al. developed the first three generations of cytosine base editors (CBEs). The third generation CBE, BE3, is composed of the naturally occurring cytosine deaminase rAPOBEC1, coupled with a SpCas9 nickase (SpCas9n) and a uracil glycosylase inhibitor (UGI). APOBEC1 acts on single stranded DNA (ssDNA) and catalyses a cytosine (C) to uracil (U) transition. The UGI inhibits the endogenous base excision repair pathway that would catalyse a repair of the newly edited U. Finally, SpCas9n “nicks” the non-edited strand and therefore encourages cellular mismatch repair of the non-edited rather than the edited strand. Because U is read as a thymine (T) by the DNA polymerase during mismatch repair, CBEs are able to catalyse a **C**:G to **T**:A transitions (edited strand in bold), within a 5 bp editing window in the ssDNA bubble created by SpCas9 [2]. Developing an adenine base editor (ABE) that would enable **A**:T to **G**:C edits was particularly attractive, since C>T SNVs account for over half of human pathogenic SNV recorded in the ClinVar database and are the most common human pathogenic SNV [3]. Gaudelli et al. used the naturally occurring ecTadA as a starting point of directed evolution and developed an ABE7.10 construct that consists of a heterodimeric wtTadA-TadA* fused to a SpCas9n [4]. Both CBE and ABE have been developed further into an eighth generation of ABEs (ABE8) [5,6] and fourth generation CBE, BE4 [7]. These base editors exhibit higher levels of on-target editing activity and lower DNA and RNA off-target effects. Guide RNA (gRNA)-independent DNA off-target effects are of particular concern for CBEs [8]. Recently, a new class of base editors, Glycosylase base editors (GBE) were described. GBE are the first to enable editing of C:G to G:C transversion mutations in mammalian cell lines [9,10,11]. This is achieved by making use of the endogenous cellular base excision repair (BER) pathway and fusing a uracil DNA glycosylase (UDG) enzyme to an APOBEC deaminase. This catalyses excision of the U and creation of an abasic site followed by mutagenesis across this abasic site [9,10,11]. While we have included GBE in this analysis, DNA off-target effects as well as the reason behind different editing activities across target sites are still outstanding for these constructs.

Similar to the directed evolution of deaminases, the Cas9 proteins utilised in base editors have been expanded to include Cas9 homologues from different species as well as novel, evolved Cas9 variants that feature more relaxed protospacer adjacent motif (PAM) sites, differently sized and positioned editing windows, and different sizes and therefore different packaging capabilities, have been developed. For example, the targeting scope of the *Staphylococcus aureus* Cas9 homologue SaCas9 with a PAM 5′-NNGRRT-3′ was increased up to four-fold with the evolution of triple mutant variant SaCas9-KKH, which features the more relaxed PAM site 5′-NNGRRT-3′ [12]. SaCas9 and its variants are particularly attractive Cas proteins because they can be packaged into an adeno-associated virus (AAV), which has become the primary vector for retinal gene therapy [13,14,15]. Similarly, the SpCas9 variants xCas and SpCas9-NG recognize a permissive, widely available 5′-NG-3′ PAM site [16,17].

The Cas platform was expanded with the addition of Cas12, which is unique in its capability to recognise T-rich PAM sites upstream of the target base at the 5′-end of the protospacer motif. Both the Cas12a variants of *Lachnospiraceae bacterium* (LbdCas12a) as well as *Acidaminococcus* sp. AsdCas12a were shown to efficiently edit in mammalian cell lines when coupled to both an ABE and a CBE [18,19]. Recently, a mutant variant of the Cas12f family, which is less than half the size of SpCas9, was reported to function in mammalian cell line. CasMINI has the same PAM as LbCas12a, but features a very restrictive editing window, which makes the search for appropriate PAM sites more challenging but limits the possibility of bystander edits [20].

The *CRB1* gene is located on 1q31.3 and encodes a highly conserved transmembrane protein in retina and brain of humans and other mammals [21,22]. Biallelic pathogenic *CRB1* variants have been linked to two severe, early-onset forms of retinal dystrophy: they have been found to cause 3–9% of autosomal recessive retinitis pigmentosa (RP) cases and 7–17% of autosomal recessive Leber congenital amaurosis (LCA) [23,24,25]. The most characteristic morphologic hallmark of *CRB1*-associated retinal degeneration is the thickening of the retina, which stands in contrast to other molecular forms of RP or LCA, in which the inner retina progressively thins due to photoreceptor (PR) loss [26].

**Figure 1:**
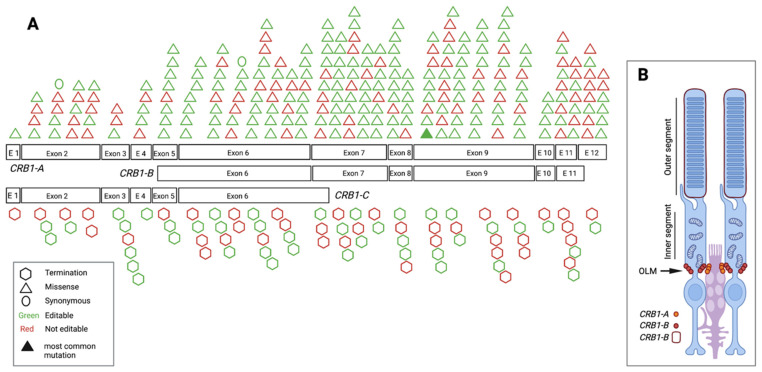
Schematic drawing of pathogenic, exonic single nucleotide variants (SNVs) along the CRB1 isoforms. (**A**) *CRB1* has three retinal isoforms, from which CRB1-B is the most highly expressed overall. The most commonly reported pathogenic/likely pathogenic/conflicting pathogenic SNV across both datasets, c.2843G>A, p.Cys948Tyr, can be found in the first nucleotide of exon 9. (**B**) CRB1-B is found in photoreceptors (PR), whereas CRB1-A is found in Müller glia cells (MGCs).

A molecular hallmark of human retinal *CRB1* expression is that apart from the constitutive isoform, *CRB1-A*, two additional isoforms, the *CRB1-B* and *CRB1-C*, are expressed at meaningful levels and differ enough in their sequence to encode a functional difference on a protein level [27]. The expression of these isoforms appears to be cell type specific. CRB1-A is expressed in the subapical region of Müller glia cells (MGCs), while CRB1-B is expressed in PRs [27]. The protein’s constitutive isoform, CRB1-A, has a large extracellular domain made up of 19 epidermal growth factor (EGF)-like and three laminin A-like domains, as well a highly conserved transmembrane domain followed by a short intracellular domain that contains FERM/PDZ binding motifs [23]. CRB1-B has a unique C- and N-terminus but shares the transmembrane domain as well as a large part of the extracellular domain with CRB1-A, while CRB1-C shares the first six exons with CRB1-A (Figure 1).

Currently, there are no therapies available for *CRB1*-associated IRDs. Gene therapy utilizing AAVs as vectors for retinal transgene delivery has established itself as a safe and efficacious treatment for recessive and X-linked pathogenic mutations in humans [13,28,29]. Nonetheless, a gene supplementation approach for *CRB1*-associated IRDs is inherently limited by its capability to only supplement one of the three *CRB1* isoforms. Indeed, proof of principle studies for the treatment of *CRB1*-induced retinal degeneration have focused on delivering *CRB2* to rescue a *CRB1*-induced phenotype in murine models [30,31]. With its ability to irreversibly correct point mutations by chemical modification of nucleobases, base editing is becoming an increasingly viable therapeutic option for the treatment of genetic diseases not amenable to a gene supplementation with AAV. Furthermore, previous analysis has shown that pathogenic SNVs editable with CRISPR/Cas commonly occur in the five large recessively inherited IRD genes *ABCA4*, *CDH23*, *USH2A*, *MYO7* and *EYS* [32].

By evaluating the Leiden open-source variation database (LOVD) and ClinVar database, this review aims to (1) characterise *CRB1* variants and (2) evaluate the amenability of pathogenic SNVs in the *CRB1* gene to base editing through the identification of appropriate base editor and PAM sites for each pathogenic SNV. This will enable an indication of the feasibility and therapeutic need of gene editing for *CRB1*-associated retinal degeneration.

## 2. Materials and Methods

All *CRB1* variants were downloaded from the LOVD and ClinVar database on 25 September 2021. Duplicates within datasets were removed and entries that had been flagged by the database due to incorrectness were excluded from analysis. Entries were then merged between datasets and those present in both databases were combined. Each variant was labelled according to clinical significance stated in the database entries: benign, likely benign, benign/likely benign, uncertain significance, pathogenic, likely pathogenic, pathogenic/likely pathogenic, and unclassified. In accordance with the American College of Medical Genetics (ACMG) guidelines, variants with conflicting interpretations within or between databases were reported between three levels of pathogenicity: benign or likely benign vs. uncertain significance vs. pathogenic or likely pathogenic. This means that a variant was only labelled as conflicting if its classification differed between these three categories. For example, a variant that was labelled as both benign and likely pathogenic was labelled as conflicting whereas an SNV labelled as both pathogenic and likely pathogenic was not labelled as conflicting. In addition to pathogenic and likely pathogenic variants, conflicting variants whose label included at least one pathogenic classification were included in the analysis (while retaining their conflicting classification). If an SNV present in both datasets was classified in one, but unclassified in the other dataset, the variant was labelled according to the existing classification.

All pathogenic, likely pathogenic and conflicting pathogenic variants were labelled by variant consequence: missense, nonsense, synonymous, insertion/deletion/duplication, splice, intronic and copy number variation (CNVs). A nonsense variant was defined as a SNV resulting in a stop codon, splice was defined as a variant disrupting a canonical splice donor (+1 and +2) or acceptor (−2 and −1) site, and intronic was defined as a variant occurring between the intronic +3 to −3 positions. The SNVs were then labelled as a transition or transversion SNVs based on their nucleic acid change. Transitions were defined as four possible purine to purine or pyrimidine to pyrimidine changes (A>G, T>C, C>T and G>A), while transversions were defined as 12 possible purine to pyrimidine or pyrimidine to purine changes (C>G, G>C, A>T, T>A, G>T, T>G, C>A, A>C, T>G, G>T, A>G and G>A). Each transition or transversion was further classified by its amenability to correction with a currently available base editor (an ABE, CBE or GBE). Pathogenic, exonic SNVs were mapped along exonic sequence of *CRB1*.

**Figure 2:**
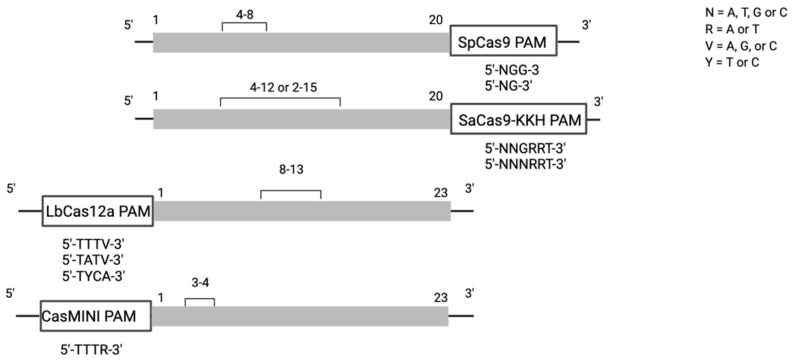
Overview of Cas proteins, their PAM sites and editing windows evaluated in this analysis. Whereas the Cas9 family has a PAM sequence downstream of the target base and targets G-rich sequences, the Cas12 family targets T-rich PAM sites and the PAM is positioned upstream of the target base. CasMINI has a small editing window, whereas SaCas9 has the largest editing window.

Lastly, the editable pathogenic SNVs were analysed for adjacent PAM sites that would allow for sequence recognition by a Cas protein. The PAM sites of the following select Cas proteins were investigated: SpCas9, xCas/SpCas-NG as well as the Cas9 variants SaCas9 and SaCas9-KKH. From the Cas12 family, LbCas12a, AsCas12a as well as the recently described compact CasMINI were included in the PAM site analysis. In an effort to make the analysis translationally focused, only Cas proteins that have been successfully edited in a mammalian cell line when coupled to the required BE were taken into consideration. Since evidence for the functionality of CasMINI has been currently shown only when coupled with an ABE, only SNVs amenable to correction by an ABE were considered for CasMINI PAM sites. Conversely, since GBE have solely been coupled with SpCas9 as well as the SpCas9 homologue SpCas9-NG, only PAM sites for these Cas9 proteins were considered for variants amenable to a GBE. For an overview of the Cas proteins and their PAM sites evaluated in this analysis, please see Figure 2 as well as Table 1.

## 3. Results

### 3.1. Characterisation of Leiden Open-Source Variation Database (LOVD) and ClinVar Database

**Figure 3:**
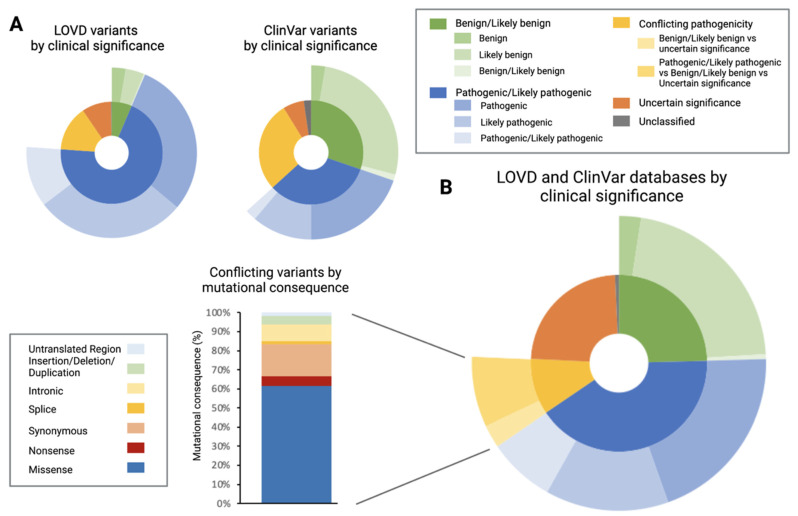
Breakdown of variants found in the Leiden open-source variation database (LOVD) and ClinVar database. (**A**) The ClinVar database exhibited a much higher proportion of benign/likely benign variants. (**B**) When the two datasets were merged, 41% were pathogenic/likely pathogenic variants and 11% were labelled as conflicting (reported on three levels of pathogenicity). Of the conflicting variants, over 60% were missense and almost 20% were synonymous variants.

The ClinVar database contained almost double the variants listed in LOVD (*n* = 876, *n* = 450, respectively). The distribution of clinical significance between datasets varied markedly (Figure 3A). The LOVD identified 6% of *CRB1* variants to be benign/likely benign, whereas this category made up 30% of variants reported in ClinVar, a percentage that was nearly equal to the 33% of pathogenic/likely pathogenic variants reported in this database. In contrast, 70% of the variants in the LOVD were classified as pathogenic/likely pathogenic. Fourteen percent of variants in the LOVD were of uncertain significance, whereas this category made up 28% of the variants in the ClinVar database. The percentage of unclassified and conflicting variants was similar between the two databases. A total of 1115 individual variants were present after both datasets had been merged (Figure 3B).

Of these, 41% were pathogenic/likely pathogenic and 11% of variants labelled as having conflicting interpretations. However, 73% of these conflicting variants had been labelled as pathogenic in at least one instance (accounting for 8% of total mutations). Of the total number of variants with conflicting interpretations, most (61%) were missense mutations, with the second largest group being synonymous mutations.

From the total number of variants, 211 were reported in both datasets, whereas the remaining 904 variants could be found either only in the LOVD or ClinVar. Of the 211 variants reported in both datasets, 52% were pathogenic/likely pathogenic, while 35% were labelled as having conflicting interpretations. Moving forward, pathogenic/likely pathogenic as well as variants with conflicting interpretations that had been labelled pathogenic in at least one instance (conflicting pathogenic) were included in the analysis. For simplicity’s sake, they will be referred to as “pathogenic/likely pathogenic/conflicting pathogenic SNVs” hereafter. Combined, pathogenic/likely pathogenic/conflicting pathogenic SNVs made of 49% (n = 521) of the SNVs of the total dataset.

### 3.2. Pathogenic/Likely Pathogenic/Conflicting Pathogenic Single Nucleotide Variants (SNVs) by Mutational Consequence

**Figure 4:**
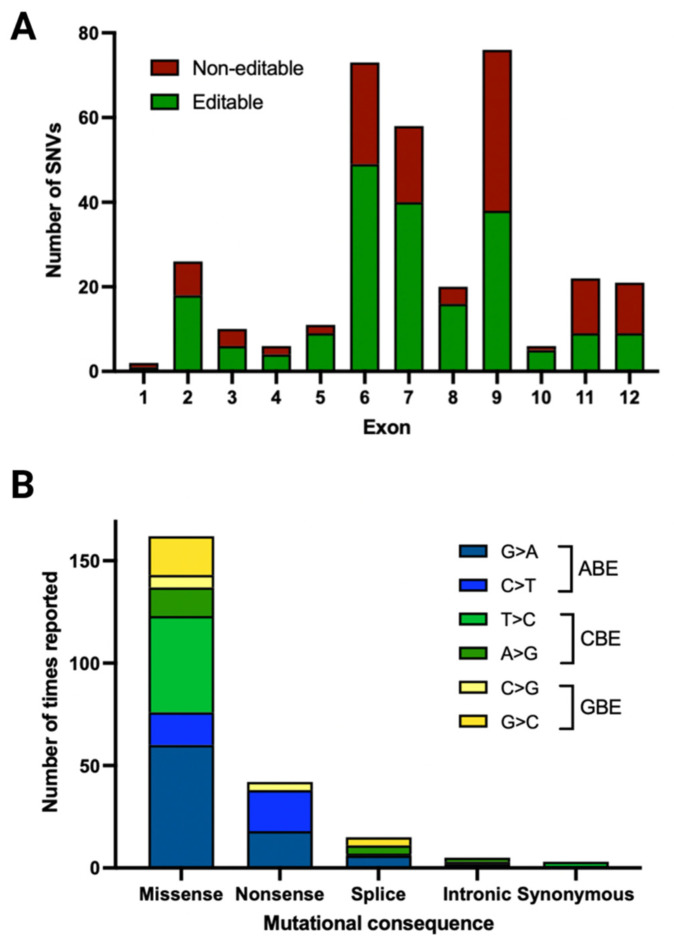
Pathogenic SNVs by exon location and editability as well as mutational consequence. (**A**) 57% of pathogenic SNVs are present in exon 6, 7 and 9, which corresponds to the length of these exons relative to the entire coding sequence (cds). Of the pathogenic SNVs in exon 6, 67% are editable. (**B**) The most common pathogenic, editable SNV is a missense mutation, followed by nonsense and splice site mutations.

The three most common mutational consequences in the pathogenic/likely pathogenic/conflicting pathogenic SNVs were missense (47%), insertion/deletions/duplications (30%) and nonsense (16%) consequences. Splice and intronic mutations made up 8% of the variants (6% and 2% respectively), while synonymous mutations made up <1% of the dataset (Figure 4B).

### 3.3. Pathogenic/Likely Pathogenic/Conflicting Pathogenic Exonic SNVs and Their Location

When mapping the pathogenic/likely pathogenic/conflicting pathogenic SNVs along the exonic *CRB1-A* sequence as well as its two most important retinal isoforms, *CRB1-B* and *CRB1-C*, it became apparent that 57% of pathogenic/likely pathogenic/conflicting pathogenic SNVs were present in exon 6, 7 and 9 (Figure 1 and Figure 4A). This is in keeping with the fact that exons 6, 7, and 9 take up 57% of the exonic coding sequence (2448 bp out of 4221 bp) of *CRB1-A*. Exon 6 is shared among all isoforms and pathogenic/likely pathogenic/conflicting pathogenic SNVs recorded in this isoform account for 21% of pathogenic SNVs. Of the pathogenic/likely pathogenic/conflicting pathogenic SNVs in exon 6, 67% were editable.

### 3.4. Pathogenic/Likely Pathogenic/Conflicting Pathogenic SNVs by Times Reported

**Figure 5:**
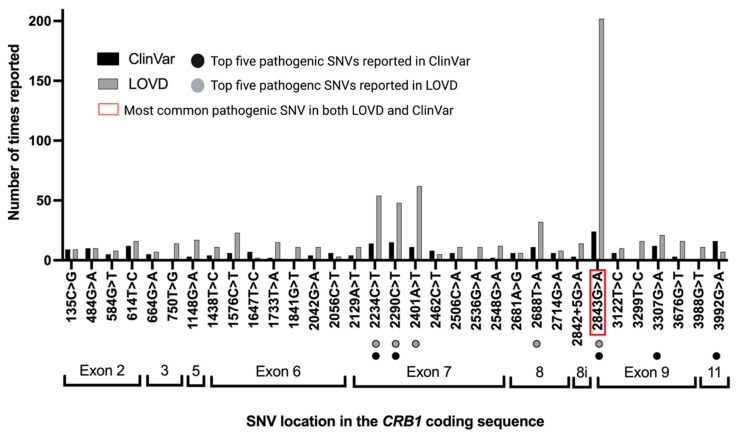
Most commonly reported CRB1 mutations by frequency. The most frequently reported mutation in both the LOVD and ClinVar databases was c.2843G>A. Furthermore, c.2234C>T and c.2290C>T were among the top five most commonly reported pathogenic SNVs in both datasets. Of the pictured mutations, 70% would be targetable by a BE, with an ABE alone being able to target 36% of these mutations.

In the LOVD, 153 out of 282 (54%) pathogenic/likely pathogenic/conflicting pathogenic SNVs were reported two or more times. In the ClinVar database, 94 pathogenic/likely pathogenic/conflicting pathogenic SNVs out of 238 (39%) were reported two or more times. Figure 5 shows the top 23 reported mutations in the LOVD and the top 22 reported mutations from each database side by side. Eighteen out of these mutations were labelled as conflicting (pathogenic/likely pathogenic vs. benign or uncertain significance). This includes c.2843G>A, p.Cys948Tyr, the most commonly reported SNV across both datasets. This was recorded 24 times in the ClinVar dataset and 202 times in the LOVD. Furthermore, c.2234C>T and 2290C>T were also commonly reported pathogenic/likely pathogenic/conflicting pathogenic SNVs in both datasets.

### 3.5. Pathogenic/Likely Pathogenic/Conflicting Pathogenic SNVs and Their Amenability to Base Editing

**Figure 6:**
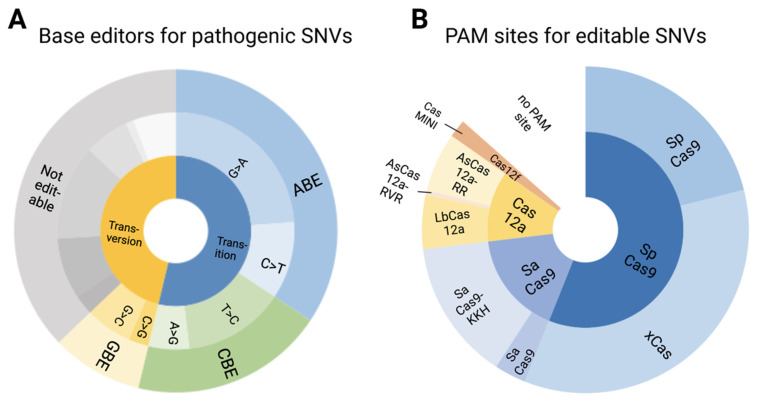
Amenability of pathogenic SNVs to base editing. (**A**) 53% of pathogenic SNVs were transitions and therefore amenable to base editing. 9% of editable SNVs were C:G to G:C transversions, editable with GBEs. In total, 62% of pathogenic SNVs were amenable to a base editor. The largest subset of editable SNVs (34%) contained G:C to A:T mutations and was thus amenable to treatment with an ABE. (**B**) Of the editable SNVs, 87% were found to have a PAM site in the appropriate editing window. The largest proportion were amenable to SpCas9, in particular the Cas9 variant xCas, which is due to the relaxed PAM site of 5′-NG-3′. The novel CasMINI bears great potential due to its small size but was not amenable to many SNVs due to the small editing window and specific PAM requirements.

Although prime editing has the potential for correcting insertion/deletions/duplications [38], currently available base editors are limited to correcting SNVs. In this dataset, SNVs made up 69% of pathogenic/likely pathogenic/conflicting pathogenic variants, of which a little over half (53%) were transitions, while the remainder were transversions (Figure 6A). All transitions were labelled as editable, while 34% of transversions were G:C to C:G transversions and therefore potentially amenable to editing with GBEs. In total, 62% of pathogenic/likely pathogenic/conflicting pathogenic SNVs are amenable to base editing with the largest proportion (34%) of editable SNVs being G:C to A:T mutations. Almost half as many pathogenic/likely pathogenic/conflicting pathogenic SNVs (19%) are amenable to a CBE, while 9% are amenable to a GBE. G>A mutations represented the largest subset pathogenic/likely pathogenic/conflicting pathogenic SNVs (23%), followed by T>C and C>T (14% and 11%, respectively). Of the most commonly reported pathogenic/likely pathogenic/conflicting pathogenic SNVs in both datasets, 70% would be targetable by a base editor, with an ABE alone being able to target 36% of the most commonly reported pathogenic/likely pathogenic/conflicting pathogenic SNVs.

### 3.6. Editable Pathogenic SNVs and the Availability of PAM Sites

While a mutation may be editable, the presence of a PAM site is necessary to ensure recognition by the Cas protein coupled to selected base editor. From the pathogenic/likely pathogenic/conflicting pathogenic SNVs amenable to a BE, 87% were found to have a PAM site in the appropriate editing window (Figure 6B). SpCas9 and its variants accounted for 56% of PAM sites, with SpCas9 having a PAM site in 21% of cases and xCas in 35% of cases. SaCas9 and its more relaxed SaCas9-KKH variant accounted for nearly 17% of PAM sites (3% and 14%, respectively). The Cas12a family was able to cover 12% of PAM sites, while the novel CasMINI base editor was able to cover 2% of pathogenic/likely pathogenic/conflicting pathogenic SNVs. 13% of editable pathogenic/likely pathogenic/conflicting pathogenic SNVs did not have a PAM site.

## 4. Discussion

In summary, 69% of total SNVs were classified as pathogenic/likely pathogenic/conflicting pathogenic SNVs across the LOVD and ClinVar database. ABEs were the most commonly required base editors and have the potential of targeting 34% of pathogenic/likely pathogenic/conflicting pathogenic SNVs. In total, 62% of pathogenic/likely pathogenic/conflicting pathogenic SNVs were amenable to currently available base editors. Of the pathogenic/likely pathogenic/conflicting pathogenic SNVs targetable with a DNA base editor, 87% had a PAM site that put the target mutation within the appropriate window of editing of currently available Cas proteins. When selectively looking at the 22 most frequently reported pathogenic/likely pathogenic/conflicting pathogenic SNVs across both datasets, 70% were targetable with a base editor. The most common pathogenic/likely pathogenic/conflicting pathogenic SNVs according to the analysed datasets was c.2843G>A, which is targetable with an ABE.

### 4.1. Discrepancy between Databases

Both the LOVD and ClinVar database are curated gene variant databases, also known as locus-specific databases (LSDB), that store information on the variants of the human genome and their phenotypic consequences [39]. LOVD was founded 15 years ago, ClinVar in 2012. The ClinVar dataset contained more benign mutations than the LOVD (30% vs. 6%). It is possible that this might be due to the difference in submitter profiles between the two databases. The marked discrepancy between the frequency of individual mutations being reported might be due to overreporting of variants from patient mutations that have been included in more than one published dataset. When looking at variants with conflicting interpretation by consequence, missense mutations are the most common as their consequence is particularly difficult to assess. The sparse overlap of mutations present in both databases should be noted when searching public databases for variants.

### 4.2. Mutational Consequences of Variants

There are two synonymous variants reported in the pathogenic/likely pathogenic/conflicting pathogenic SNVs dataset, c.93C>T, p.Asn30=, and c.1647C>T, p.Asn549=. The first mutation, c.93C>T, was described only in the LOVD and labelled as pathogenic based on a publication by Li et al. in 2009 [40]. The other synonymous SNV, c.1647C>T, was present in both databases and labelled as being of conflicting interpretation. A likely pathogenic label was given to this mutation just once in a publication by Lotery et al. in 2001 [41]. It is important that both these labels were given before the publication of the American College of Medical Genetics (ACMG) guidelines [42]. Since the gold standard ACMG guidelines for variant classification were published in 2015, a review of mutations assessed before that time point may greatly improve the dataset quality and unify the classification criteria applied for mutation within and between datasets.

It is unsurprising the pathogenic/likely pathogenic/conflicting pathogenic SNVs amenable to an ABE make up the highest proportion of total pathogenic SNPs, since almost half of reported human pathogenic SNPs are C:G to T:A mutations [3,4,43]. Spontaneous hydrolytic deamination of cytosine to uracil occurs approximately 100–500 times per day in each human cell, which is likely a contributing factor to this high rate of C:G to T:A pathogenic SNVs [43]. When looking at the five most commonly reported mutations in the dataset, three of them are C>T and two are G>A transitions.

### 4.3. Further Considerations

While mutation type and presence of a PAM site are the first steps in determining the suitability of a pathogenic/likely pathogenic/conflicting pathogenic SNV for base editing, bystander edits within the editing window as well as genome wide RNA off-target should also be evaluated. While a large editing window, such as that of SaCas9-KKH, increases the probability of being able to target a given variant, it also increases the chance of bystander edits. For example, the pathogenic missense variant c.3074G>A, p.Ser1025Asn, lies at position 10 of the editing window of SaCas9-KKH. There are three further As in the editing window at positions 6, 8 and 9 which have a high likelihood of being targeted by the chosen ABE as well due to the inherent (albeit variable) processivity of BEs. This would result in missense mutations, which would require evaluation in silico and functionally for pathogenicity. If, hypothetically, this same mutation were at position 3 of the editing window of CasMINI, whose 2 bp editing window only covers position 3 and 4, the likelihood of bystander mutations would be very low, since there is no additional A at position 4. On the other hand, Cas proteins with a small editing window (such as SpCas9 or CasMINI) may lead to a smaller than expected proportion of amenable mutations, even with a ubiquitous PAM requirement such as that of SpCas9 (5′-NGG-3′). In our data set, 21% of pathogenic/likely pathogenic/conflicting pathogenic SNVs were found to have an SpCas9 PAM site within range of the editing window, which is in concordance with research that suggest 26% of SNVs will fall within the editing window of an SpCas9 PAM site [16]. Ideally, a large Cas library would feature Cas proteins with small editing windows but a large variety of PAM sites to choose from. When thinking about translational benefit, another factor to consider is the 4.7kb packaging capacity of an AAV vector [44,45]. SpCas9 is the largest Cas9 protein (4096 bp; 1368 aa) and packaging it into an AAV with a BE and a gRNA is not possible. Even without the gRNA or regulatory sequences, the packaging size of a BE containing a SpCas9 is 5.2 kb [46]. Two approaches have been used to circumvent this challenge: Levy et al. optimised a split base-editor dual AAV vector system to deliver ABE and CBE in mice. The split BEs are reconstituted by trans-splicing inteins and showed therapeutically relevant efficiencies in a variety of organs including the retina [46]. Another option circumventing the packaging limitation of AAV is to use a lentiviral vector with a packaging capacity of approximately 8kb [47]. This approach was used by Suh et al. to treat *Rpe65*-ssociated IRD in a *rd12* murine model [35]. While lentiviral vectors transduce RPE cells, it transduces PRs poorly [48], which limits the use of these vectors. This discussion highlights the potential of small Cas orthologs such as CasMINI that would allow an all-in-one packaging approach of gRNA, base editor and Cas protein in a single AAV vector [49]. In addition to being half the size of SpCas9 (529aa), CasMINI [20] has the additional benefit of a small editing window that would greatly limit bystander mutations.

Due to the autosomal recessive nature of the *CRB1*-associated RP disease, it is presumed that correction of one allele is sufficient to rescue disease phenotype. Unlike in other recessive, IRD causing genes such as *ATP-binding cassette 4*-associated (*ABCA4*), where complex alleles present a challenge [50] and multiplexed CRISPR technologies might be needed for phenotype correction [51], complex alleles are not widely described in *CRB1* patients. As with gene replacement therapy, questions about percentage of transgene correction needed to rescue phenotype, remain to be answered.

When thinking about translational therapeutic potential, the immune response to AAV as the predominant viral vector in current gene therapy trials should be taken into consideration: as seen in both clinical gene therapy phase 1/2 dose escalation trials for LCA, choroideremia and *Retinitis Pigmentosa GTPase Regulator* (*RPGR*)-associated X-linked RP [13,15,52], as well as in pre-clinical murine and primate gene therapy trials [53,54], AAV leads to a dose-dependent innate and adaptive immune response in the eye despite the perceived low immunogenicity of these viral vectors and the relatively immune privilege of the eye.

## 5. Conclusions

Due to multiple functional isoforms, *CRB1*-associated retinal degeneration poses a unique therapeutic challenge that is unlikely to be addressed by gene replacement therapy. Since 62% of pathogenic/likely pathogenic/conflicting pathogenic SNVs are amenable to correction with a base editor and 87% of these SNVs were found to have a suitable PAM site, gene editing represents a promising therapeutic avenue for *CRB1*-associated retinal degenerations.

## Figures and Tables

**Table 1:** Overview of Cas proteins, their PAM sites and editing windows evaluated in this analysis. For a comprehensive overview of published Cas proteins coupled to base editors (BEs) see Kantor et al. [33]. R = G or A; V = A, G or C; Y = C or T.

Cas Family			PAM Sequence 5’-3’	PAM Location Relative to Target Base	Editing Window =	Previously Coupled with BE Class	Size (in aa)
Cas9		SpCas9	NGG	downstream	4–8	ABE7.10 [4], ABE8 [6], ABEmax [34] BE3 [2], BE4 [7], BE4max [34] GBE [9]	1368
		xCas/SpCas-NG*	NG	downstream	4–8	ABE7.10 [16,35] BE3 [16] GBE* [9]	
		SaCas9	NNGRRT	downstream	4–12	ABE7.10, ABE8 [6] BE3 [36], BE4 [7]	1053
		SaCas9-KKH	NNNRRT	downstream	2–15	ABE7.10 [37], ABE8 [6] BE3 [36]	
Cas12	Cas12a	LbCas12a	TTTV	upstream	8–13	ABE8 [6] BE3 [19]	1228
		enAsCas12a-RR enAsCas12a-RVR	TATV, TYCV	upstream	8–13	ABE8 [6] BE3 [19]	
	Cas12f	CasMINI	TTTV	upstream	3–4	ABE8 [20]	529

## Data Availability

The data analyzed are from publicly available sources. All other data are included in the manuscript.
